# Maternal Fasting Plasma Glucose Level in Early Gestation and Developmental Delay in 2-year-old Children

**DOI:** 10.1210/clinem/dgae825

**Published:** 2025-01-20

**Authors:** Chikana Kawaguchi, Mami Ishikuro, Ryota Saito, Keiko Murakami, Aoi Noda, Genki Shinoda, Misato Aizawa, Hisashi Ohseto, Noriyuki Iwama, Masatsugu Orui, Taku Obara, Shinichi Kuriyama

**Affiliations:** Department of Molecular Epidemiology, Graduate School of Medicine, Tohoku University, Sendai, Miyagi 980-8575, Japan; Department of Molecular Epidemiology, Graduate School of Medicine, Tohoku University, Sendai, Miyagi 980-8575, Japan; Tohoku Medical Megabank Organization, Tohoku University, Sendai, Miyagi 980-8573, Japan; School of Medicine, Tohoku University, Sendai, Miyagi 980-8575, Japan; Tohoku Medical Megabank Organization, Tohoku University, Sendai, Miyagi 980-8573, Japan; Department of Molecular Epidemiology, Graduate School of Medicine, Tohoku University, Sendai, Miyagi 980-8575, Japan; Tohoku Medical Megabank Organization, Tohoku University, Sendai, Miyagi 980-8573, Japan; Department of Pharmaceutical Sciences, Tohoku University Hospital, Sendai, Miyagi 980-8574, Japan; Department of Molecular Epidemiology, Graduate School of Medicine, Tohoku University, Sendai, Miyagi 980-8575, Japan; Tohoku Medical Megabank Organization, Tohoku University, Sendai, Miyagi 980-8573, Japan; Department of Molecular Epidemiology, Graduate School of Medicine, Tohoku University, Sendai, Miyagi 980-8575, Japan; Department of Molecular Epidemiology, Graduate School of Medicine, Tohoku University, Sendai, Miyagi 980-8575, Japan; Tohoku Medical Megabank Organization, Tohoku University, Sendai, Miyagi 980-8573, Japan; Department of Obstetrics and Gynecology, Tohoku University Hospital, Sendai, Miyagi 980-8574, Japan; Department of Molecular Epidemiology, Graduate School of Medicine, Tohoku University, Sendai, Miyagi 980-8575, Japan; Tohoku Medical Megabank Organization, Tohoku University, Sendai, Miyagi 980-8573, Japan; Department of Molecular Epidemiology, Graduate School of Medicine, Tohoku University, Sendai, Miyagi 980-8575, Japan; Tohoku Medical Megabank Organization, Tohoku University, Sendai, Miyagi 980-8573, Japan; Department of Pharmaceutical Sciences, Tohoku University Hospital, Sendai, Miyagi 980-8574, Japan; Department of Molecular Epidemiology, Graduate School of Medicine, Tohoku University, Sendai, Miyagi 980-8575, Japan; Tohoku Medical Megabank Organization, Tohoku University, Sendai, Miyagi 980-8573, Japan; Department of Disaster Public Health, International Research Institute of Disaster Science, Tohoku University, Sendai, Miyagi 980-8573, Japan

**Keywords:** children, developmental delay, pregnancy, fasting plasma glucose level, Japan

## Abstract

**Background:**

The association of maternal hyperglycemia with childhood developmental delay has been examined; however, only 2 studies used maternal blood glucose level as a continuous variable as an exposure. A present study aimed to investigate the influence of maternal fasting plasma glucose (mFPG) level in early gestation on developmental delay in children.

**Methods:**

This cohort study included 1541 mother–child pairs who participated in the Tohoku Medical Megabank Project Birth and Three-Generation Cohort Study. mFPG level before 24 gestational weeks was obtained as a continuous and categorical variable. Developmental delay in 2-year-old children was assessed by mothers using the Ages and Stages Questionnaire (third edition). Associations between mFPG level and developmental delay in children were evaluated using multiple logistic regression analyses.

**Results:**

The prevalence of mFPG level ≥95 mg/dL was 5.2%. At 2 years old, 15.1% of the children had developmental delays. mFPG level as a continuous variable was not associated with an increased risk of developmental delay across the 5 domains in children [adjusted odds ratio (aOR), 95% confidence interval (CI): 1.004, 0.990-1.018]. mFPG level ≤70 mg/dL was associated with developmental delay across 5 domains (aOR, 95% CI: 0.464, 0.229-0.943) in children than that with a mFPG level 71 to 94 mg/dL. No association was found between mFPG level ≤70 mg/dL and ≥95 mg/dL and developmental delay in any domains among children.

**Conclusion:**

mFPG level in early gestation was not associated with an increased risk of any developmental delays in 2-year-old children.

Developmental delay is defined as a lag in acquiring communication, social, and daily living skills in children compared with those of age-matched peers (1). Some of these children may be at risk of developing behavioral or developmental disorders, such as autism spectrum disorder (ASD) (2). Globally, the prevalence of ASD and attention deficit hyperactivity disorder (ADHD) in children under 5 years of age is approximately 0.4% and 0.2%, respectively (3). A study conducted in Hirosaki, Japan, reported that the adjusted prevalence of ASD was 3.2% in children aged 5 years (4). As a consequence of their unique ways of expressing themselves, children may experience interpersonal problems (5). Thus, the American Academy of Pediatrics emphasizes the importance of early detection of developmental disorders and recommends screening for developmental delay using standardized tests in children below the age of 3 years (2).

Several risk factors for developmental disorders have been considered (6). Systematic reviews and meta-analyses have shown that pregestational diabetes mellitus (DM) and gestational diabetes mellitus (GDM) are possible risk factors for ASD in offspring (6, 7), neurocognitive and behavioral outcomes (8), and developmental delay (9, 10). These previous studies mainly obtained a history or a diagnosis of pregestational DM or GDM as an exposure (6-10) and were not focused on the maternal blood glucose level, which is usually measured at the prenatal health check-ups (11). To the best of our knowledge, only 2 previous studies have examined the association between maternal blood glucose levels and neurodevelopmental outcomes in children (12, 13). Wang et al obtained maternal fasting plasma glucose (mFPG) levels between 24 and 28 weeks when GDM is diagnosed (11), which was associated with an increased risk of developmental delay in communication and personal-social domains in children (12). Chen et al obtained maternal random capillary glucose levels throughout pregnancy and found that high random capillary glucose levels in early pregnancy were associated with ASD and ADHD (13). However, the influence of maternal blood glucose level in early pregnancy on developmental delay in children is still unknown. It is known that the first to second trimester are significant periods for fetal brain development (14), and a study reported the need to examine the association between GDM diagnosed before 26 weeks and developmental delay in children (15). A randomized controlled trial reported that treatment of GDM diagnosed before 20 weeks of gestation decreased an incidence of a composite of adverse neonatal outcomes compared with no treatment (16). In Japan, approximately 14 prenatal health check-ups are provided to all pregnant women throughout pregnancy, and approximately 90% of municipalities conducted the blood glucose test in early pregnancy (17) to detect overt diabetes (11, 18). GDM diagnosis is recommended between 24 and 28 weeks of gestation (11, 18). Thus, blood glucose level in early gestation is a clinically important measurement until GDM diagnosis is conducted, and early gestation may be also a significant period for neurodevelopment in children.

Therefore, the present study aimed to examine the association of mFPG level in early gestation, as both a continuous and categorical variable, with developmental delay in children at 2 years of age.

## Materials and Methods

### Study Population

**Figure 1.**
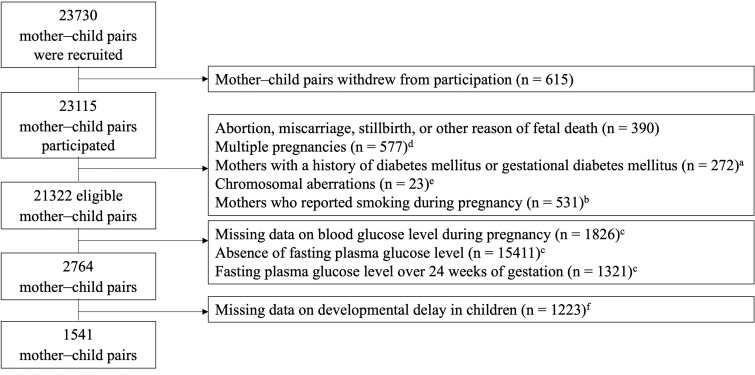
Flow diagram of participants in this study. ^a^Data on maternal history of diabetes mellitus and gestational diabetes mellitus were obtained from medical records during early pregnancy. ^b^Data on maternal smoking status was obtained from the questionnaire during early pregnancy. ^c^Data on maternal blood glucose levels and dates of measurements were obtained from medical records of prenatal health check-ups. ^d^Data on multiple pregnancies was obtained from medical records at delivery and hospitalization. ^e^Data on chromosomal aberrations in children were obtained from the questionnaire at the age of 1 month. ^f^Data on developmental delay in children were obtained from a questionnaire at the age of 2 years.

Data were collected from the Tohoku Medical Megabank Project Birth and Three-Generation Cohort Study (TMM BirThree Cohort Study) (19, 20). The TMM BirThree Cohort Study protocol was reviewed and approved by the Ethics Committee of the Tohoku University Tohoku Medical Megabank Organization (2013-1-103-1), and all methods in our study were performed in accordance with the Declaration of Helsinki (21). All participants provided informed consent at enrollment. In this cohort study, pregnant women and their families were recruited between 2013 and 2017 at obstetric clinics and hospitals in Miyagi Prefecture, Japan. Consequently, 32 968 eligible pregnant women were contacted, and 23 406 pregnant women with 23 730 fetuses were included. Exclusion criteria were (1) withdrawal from participation; (2) abortion, miscarriage, stillbirth, or other causes of fetal death; (3) multiple pregnancies; (4) mothers with a history of DM or GDM; (5) chromosomal aberrations in children; and (6) mothers who reported smoking during early pregnancy. Of the 23 730 mother–child pairs, 2408 were excluded based on the aforementioned exclusion criteria. Additionally, 1826 pairs with missing data on maternal blood glucose levels, 15 411 pairs with an absence of mFPG level, and 1321 pairs with mFPG level over 24 weeks of gestation were excluded. Of the remaining 2764 mother–child pairs, 1223 were excluded because of missing data on developmental delay in children. Therefore, 1541 mother–child pairs were analyzed in this study (Fig. 1). The characteristics of the participants included and excluded from the analysis are presented in Table S1 (22). Gestational weeks at measurement of mFPG level, maternal age at delivery, educational attainment, and maternal grandparental history of DM differed between the 2 populations.

### Variables

Data on initial measurements of mFPG level before 24 weeks of gestation was obtained from the medical records of prenatal health check-ups. mFPG level, as the continuous variable, was categorized into the following 3 groups: ≤ 70, 71-94 (reference), and ≥95 mg/dL. mFPG level ≥95 mg/dL was determined based on a previous study (16). A blood glucose level ≤70 mg/dL is usually treated as hypoglycemia (23).

Using the validated Japanese version of the Ages and Stages Questionnaire, third edition (ASQ-3) (24), the presence of developmental delay was assessed by the mother when the child was 2 years old. The ASQ-3 is a screening tool for children aged between 1 and 66 months that captures developmental delays in the following 5 domains: communication (babbling, vocalizing, listening, and understanding), gross motor (arm, body, and leg movements), fine motor (hand and finger movements), problem-solving (learning and playing with toys), and personal-social (solitary social play and play with toys and other children) (25). Each domain has 6 items, and each item is assigned a score of 10, 5, or 0, corresponding to yes, sometimes, or not yet, respectively. The total score ranged from 0 to 60 for each domain. Parents could omit items when they were unsure of how to respond or because of concerns about their child's performance. Scores were not calculated if 3 or more items were omitted in a given domain. In the case of 1 or 2 omitted items, an adjusted total domain score was calculated by summing the scores of the remaining items and multiplying the score by 1.2 or 1.5, respectively (25). The developmental delay in each domain was defined when a score was ≥2 SD below the mean as an instruction state (25). If 1 of the domains was defined as developmental delay, the child was considered “positive” for developmental delay (25).

The potential confounders were selected based on previous studies (12, 15). In early pregnancy and 1 year after delivery, self-reported questionnaires were used to collect data on the following variables: maternal height and weight before pregnancy; intake of folic acid supplements in early pregnancy (yes/no); maternal educational attainment (high school or lower, junior or vocational college, or university or higher); maternal grandpaternal history of DM (yes/no); and maternal grandmaternal history of DM or GDM (yes/no). Data on parity (primipara, multipara) and child's sex (male, female) were collected from the medical records. Body mass index (BMI) before pregnancy was calculated using data on maternal height and weight and categorized as <18.5, 18.5 to 24.9, and ≥25.0 kg/m².

### Statistical Analysis

The data for the characteristics of the participants were summarized using means ± SD or counts (frequencies). Differences in characteristics by mFPG level were examined using correlation analysis, *t*-tests, ANOVA for continuous variables, chi-square tests, or Fisher's exact tests for categorical variables. For ANOVA, a multiplicity adjustment using Bonferroni's or Tukey's correction was conducted. Odds ratios (ORs) with 95% confidence intervals (CIs) were calculated using multiple logistic regression analyses to explore the association between mFPG level in early gestation and developmental delay across 5 domains and of each domain in children at 2 years of age. The analyses were adjusted for maternal age at delivery, educational attainment, BMI before pregnancy, intake of folic acid supplements in early pregnancy, maternal grandpaternal history of DM, maternal grandmaternal history of DM or GDM, parity, and child's sex. Missing confounders were imputed by fully conditional specification methods using other confounders in the data. Twenty sets of copies of the data were independently analyzed in multivariate analyses and the estimates were integrated. This imputation was conducted using PROC MI and PROC MIANALYZE in SAS statistical software (26). The complete-case analysis was also conducted using same analysis and is presented in Table S2 (22).

All analyses were performed using SAS version 9.4 (SAS Inc., Cary, NC, USA). A 2-sided *P* < 0.05 was regarded as significant.

## Results

Participant characteristics are presented in Table 1. The mean mFPG level before 24 weeks of gestation was 81.8 mg/dL. Overall, 233 (15.1%) children were defined as having developmental delays across the 5 domains at the age of 2 years. Significant differences were observed in maternal BMI before pregnancy by mFPG level in both continuous and categorical variables.

**Table 1.** Characteristics of participants by maternal fasting plasma glucose level

Participants’ characteristics	Total (n = 1541) Mean ± SD/n (%)	mFPG level (mg/dL) Mean ± SD	*P*-value*^a^*	mFPG level (mg/dL), Mean ± SD/n (%)			
				≤70 mg/dL (n = 110)	71-94 mg/dL (n = 1351)	≥95 mg/dL (n = 80)	*P*-value*^b^*
mFPG level (before 24 weeks of gestation) (mg/dL)	81.8 ± 9.7	81.8 ± 9.7	NA	67.0 ± 3.6	81.5 ± 5.7	107.0 ± 17.4	<.0001
Gestational weeks at measurement of mFPG (weeks)	11.5 ± 1.8	NA	.53	11.5 ± 2.1	11.5 ± 1.7	11.5 ± 2.5	.92
Age at delivery (years)	31.9 ± 4.7	NA	.07	32.1 ± 4.2	31.8 ± 4.7	32.8 ± 4.7	.18
Educational attainment			.06				.13
High school or lower	488 (31.7)	82.4 ± 9.9		30 (27.3)	432 (32.0)	26 (32.5)	
Junior or vocational college	582 (37.8)	81.8 ± 9.7		44 (40.0)	506 (37.4)	32 (40.0)	
University or higher	310 (20.1)	80.6 ± 8.5		31 (28.2)	267 (19.8)	12 (15.0)	
Missing	161 (10.4)	82.7 ± 10.8		5 (4.5)	146 (10.8)	10 (12.5)	
BMI before pregnancy			<.0001				<.0001
<18.5 kg/m^2^	202 (13.1)	80.6 ± 8.9		22 (20.0)	171 (12.7)	9 (11.2)	
18.5-24.9 kg/m^2^	1113 (72.2)	81.4 ± 9.2		76 (69.1)	994 (73.6)	43 (53.7)	
≥25.0 kg/m^2^	196 (12.7)	85.1 ± 12.1		11 (10.0)	160 (11.8)	25 (31.3)	
Missing	30 (2.0)	84.1 ± 8.7		1 (0.9)	26 (1.9)	3 (3.8)	
Intake of folic acid supplements in early pregnancy			.60				.59
No	689 (44.7)	81.8 ± 8.9		48 (43.6)	610 (45.1)	31 (38.7)	
Yes	842 (54.6)	81.8 ± 10.3		62 (56.4)	732 (54.2)	48 (60.0)	
Missing	10 (0.7)	84.9 ± 7.2		0 (0.0)	9 (0.7)	1 (1.3)	
Maternal grandpaternal history of DM			.14				.08
No	1304 (84.6)	81.6 ± 9.5		102 (92.7)	1139 (84.3)	63 (78.8)	
Yes	96 (6.2)	83.4 ± 10.6		3 (2.7)	85 (6.3)	8 (10.0)	
Missing	141 (9.2)	82.5 ± 11.0		5 (4.6)	127 (9.4)	9 (11.2)	
Maternal grandmaternal history of DM or GDM			.35				.42
No	1328 (86.2)	81.7 ± 9.6		100 (90.9)	1162 (86.0)	66 (82.5)	
Yes	72 (4.7)	83.0 ± 8.1		5 (4.6)	62 (4.6)	5 (6.3)	
Missing	141 (9.1)	82.5 ± 11.0		5 (4.5)	127 (9.4)	9 (11.2)	
Parity			.57				.36
Nullipara	734 (47.6)	81.8 ± 10.4		54 (49.1)	635 (47.0)	45 (56.3)	
Multipara	806 (52.3)	81.8 ± 8.9		56 (50.9)	715 (52.9)	35 (43.7)	
Missing	1 (0.1)	92.0 ± NA		0 (0.0)	1 (0.1)	0 (0.0)	
Child's sex			.53				.44
Male	802 (52.0)	82.0 ± 9.7		55 (50.0)	700 (51.8)	47 (58.7)	
Female	739 (48.0)	81.7 ± 9.7		55 (50.0)	651 (48.2)	33 (41.3)	
Developmental delay at the age of 2 years: across 5 domains			.22				.11
No	1308 (84.9)	81.7 ± 9.8		101 (91.8)	1140 (84.4)	67 (83.7)	
Yes	233 (15.1)	82.5 ± 8.8		9 (8.2)	211 (15.6)	13 (16.3)	
Developmental delay at the age of 2 years: communication			.07				.33
No	1460 (94.7)	81.7 ± 9.8		107 (97.3)	1279 (94.7)	74 (92.5)	
Yes	81 (5.3)	83.5 ± 8.0		3 (2.7)	72 (5.3)	6 (7.5)	
Developmental delay at the age of 2 years: gross motor			.68				.10
No	1459 (94.7)	81.8 ± 9.7		109 (99.1)	1275 (94.4)	75 (93.7)	
Yes	82 (5.3)	82.3 ± 8.6		1 (0.9)	76 (5.6)	5 (6.3)	
Developmental delay at the age of 2 years: fine motor			.80				.41
No	1478 (95.9)	81.8 ± 9.7		108 (98.2)	1292 (95.6)	78 (97.5)	
Yes	63 (4.1)	81.5 ± 8.7		2 (1.8)	59 (4.4)	2 (2.5)	
Developmental delay at the age of 2 years: problem-solving			.55				.40
No	1474 (95.6)	81.9 ± 9.8		106 (96.4)	1289 (95.4)	79 (98.7)	
Yes	67 (4.4)	81.3 ± 7.1		4 (3.6)	62 (4.6)	1 (1.3)	
Developmental delay at the age of 2 years: personal-social			.39				.51
No	1457 (94.5)	81.8 ± 9.7		106 (96.4)	1277 (94.5)	74 (92.5)	
Yes	84 (5.5)	82.7 ± 9.7		4 (3.6)	74 (5.5)	6 (7.5)	

For ANOVAs, Bonferroni's or Tukey's correction was used to obtain *P*-values.

Abbreviations: BMI, body mass index; DM, diabetes mellitus; GDM, gestational diabetes mellitus; mFPG, maternal fasting plasma glucose; NA, not applicable.

^*a*^Obtained using correlation analysis, *t*-test, or ANOVA.

^*b*^Obtained using ANOVA, chi-square test, or Fisher's exact test.

Table 2 shows the ORs and 95% CIs for developmental delay in the children. When mFPG level was analyzed as a continuous variable, every 1 mg/dL increase in mFPG level was not associated with an increased risk of developmental delay across the 5 domains in children (crude OR, 95% CI: 1.008, 0.994-1.022). This association remained after adjusting for potential confounders [adjusted OR (aOR), 95% CI: 1.004, 0.990-1.018]. No association was found between mFPG level as a continuous variable and developmental delay in any domains among children. When analyzed as a categorical variable, mFPG level ≤70 mg/dL was associated with developmental delay across 5 domains (crude OR, 95% CI: 0.481, 0.240-0.967) in children than that with a mFPG level 71 to 94 mg/dL. This association remained after adjusting for potential confounders (aOR, 95% CI: 0.464, 0.229-0.943). mFPG level ≥95 mg/dL was not significantly associated with an increased risk of developmental delay across 5 domains (crude OR, 95% CI: 1.048, 0.569-1.933) in children than that with a mFPG level 71 to 94 mg/dL. This association remained after adjusting for potential confounders (aOR, 95% CI: 0.833, 0.442-1.571). No association was found between mFPG level ≤70 mg/dL and ≥95 mg/dL and developmental delay in any domains among children. In the complete-case analysis, only mFPG level ≤70 mg/dL was associated with developmental delay across 5 domains (aOR, 95% CI: 0.440, 0.208-0.931) (Table S2) (22).

**Table 2.** Association between maternal fasting plasma glucose level and the developmental delay in children (n = 1541)

Subgroups	Developmental delay/total, n (%)	Crude OR (95% CI)	Adjusted OR (95% CI)*^a^*
Developmental delay: across 5 domains			
Continuous (per 1 mg/dL)	NA	1.008 (0.994-1.022)	1.004 (0.990-1.018)
≤70 mg/dL (ref: 71-94 mg/dL)	9/110 (8.2)	0.481 (0.240-0.967)	0.464 (0.229-0.943)
≥95 mg/dL (ref: 71-94 mg/dL)	13/80 (16.3)	1.048 (0.569-1.933)	0.833 (0.442-1.571)
Developmental delay: communication			
Continuous (per 1 mg/dL)	NA	1.015 (0.996-1.035)	1.009 (0.989-1.030)
≤70 mg/dL (ref: 71-94 mg/dL)	3/110 (2.7)	0.498 (0.154-1.607)	0.506 (0.155-1.651)
≥95 mg/dL (ref: 71-94 mg/dL)	6/80 (7.5)	1.440 (0.606-3.421)	1.087 (0.444-2.661)
Developmental delay: gross motor			
Continuous (per 1 mg/dL)	NA	1.005 (0.983-1.027)	1.001 (0.979-1.024)
≤70 mg/dL (ref: 71-94 mg/dL)	1/110 (0.9)	0.154 (0.021-1.118)	0.150 (0.021-1.093)
≥95 mg/dL (ref: 71-94 mg/dL)	5/80 (6.3)	1.118 (0.439-2.847)	0.947 (0.364-2.465)
Developmental delay: fine motor			
Continuous (per 1 mg/dL)	NA	0.996 (0.970-1.024)	0.991 (0.963-1.020)
≤70 mg/dL (ref: 71-94 mg/dL)	2/110 (1.8)	0.406 (0.098-1.683)	0.419 (0.100-1.756)
≥95 mg/dL (ref: 71-94 mg/dL)	2/80 (2.5)	0.562 (0.135-2.341)	0.453 (0.106-1.942)
Developmental delay: problem-solving			
Continuous (per 1 mg/dL)	NA	0.994 (0.967-1.021)	0.995 (0.968-1.021)
≤70 mg/dL (ref: 71-94 mg/dL)	4/110 (3.6)	0.785 (0.280-2.198)	0.724 (0.255-2.052)
≥95 mg/dL (ref: 71-94 mg/dL)	1/80 (1.3)	0.263 (0.036-1.923)	0.233 (0.032-1.725)
Developmental delay: personal-social			
Continuous (per 1 mg/dL)	NA	1.009 (0.989-1.030)	1.003 (0.982-1.025)
≤70 mg/dL (ref: 71-94 mg/dL)	4/110 (3.6)	0.651 (0.234-1.816)	0.637 (0.225-1.802)
≥95 mg/dL (ref: 71-94 mg/dL)	6/80 (7.5)	1.399 (0.590-3.321)	1.057 (0.432-2.584)

Abbreviations: BMI, body mass index; CI, confidence interval; DM, diabetes mellitus; GDM, gestational diabetes mellitus; NA, not applicable; OR, odds ratio.

^*a*^Adjusted for maternal age of delivery, educational attainment, BMI before pregnancy, intake of folic acid supplements in early pregnancy, maternal grandpaternal history of DM, maternal grandmaternal history of DM or GDM, parity, and child's sex.

## Discussion

The present study is the first to examine the association between mFPG level in early gestation, both as a continuous and categorical variable, and the occurrence of developmental delay in children at 2 years of age. Only mFPG level ≤70 mg/dL was associated with developmental delay across 5 domains in children.

In the present study, mFPG level as a continuous variable and mFPG level ≥95 mg/dL were not associated with an increased risk of developmental delay across 5 domains and in any domains. Among 6 previous studies that examined the association between maternal DM, GDM, or FPG and developmental delay in children evaluated using ASQ-3 (12, 27-31), 2 studies reported that maternal GDM was associated with developmental delay across 5 domains (29, 30). A previous study conducted in China reported that mFPG level as a continuous variable between 24 and 28 weeks of gestation was associated with developmental delay in communication domain at the age of 12 months (adjusted relative risk, 95% CI: 1.32, 1.03-1.69) (12). Another study conducted in Finland found that GDM was associated with an increased risk of developmental delay in communication domain at the child's mean age of 42.1 months (aOR, 95% CI: 2.17, 1.28-3.66) (27). Among the 6 previous studies, no study showed a significant association between maternal DM, GDM, or FPG and developmental delay in gross motor domain among children (12, 27-31). It is consistent with the present study. Among those 6 studies (12, 27-31), a study conducted in Japan reported that maternal GDM was associated with developmental delay in fine motor domain by the age of 4 years (aOR, 95% CI: 1.24, 1.12-1.36) (28). A study conducted in Australia also reported that maternal type 2 DM and GDM was associated with developmental delay in fine motor domain among children at the age of 18 to 60 months (aOR, 95% CI: 5.30, 1.55-15.8 for type 2 DM; aOR, 95% CI: 3.96, 1.55-10.11 for GDM) (29). Regarding the problem-solving domain, 3 studies reported that maternal type 2 DM or GDM was associated with developmental delay in problem-solving domain among children (27-29). For the personal-social domain, a previous study conducted in China reported that mFPG level as a continuous variable between 24 and 28 weeks of gestation was associated with developmental delay in the personal-social domain at the age of 12 months (adjusted relative risk, 95% CI: 1.49, 1.09-2.04) (12). Another study conducted in Japan found that GDM was associated with an increased risk of developmental delay in personal-social domain by the age of 4 years (aOR, 95% CI: 1.18, 1.04-1.33) (28).

The result of the gross motor domain was consistent with the previous studies; however, other domains were not consistent with some of the previous studies. Possible reasons for the inconsistent results between the present and previous studies are, first, the difference of the measurement of exposure. The present study obtained mFPG at 1 point before 24 weeks of gestation, whereas most previous studies obtained DM or GDM, which reflects a chronic condition of hyperglycemia. According to previous studies, although pregnant women had mFPG ≥95 mg/dL—above the threshold for GDM diagnosis—in early pregnancy, 22% to 47% of them showed normal mFPG values at 24 to 28 weeks of gestation (16, 32). Thus, mFPG level can fluctuate in mid- or late pregnancy, which will influence mFPG levels on childhood developmental delay. Second, the timing of exposure was different. For example, the previous study obtained mFPG level at 24 to 28 weeks of gestation (12). Moreover, differentiation and maturation still occur in the brain after birth (33), indicating that postnatal environments also influence the development of children. Third, the timing of outcome was different. The ages of children varied between 1 month and 69 months in previous studies (12, 27-31). The cut-off value of ASQ-3 was also different in a previous study (29), which was ≥1 SD below the mean. Furthermore, ethnicity, potential confounders, and sample size may also influence the inconsistency.

Possible biological mechanisms underlying the association between a high mFPG level and developmental delay in children have been suggested. First, maternal hyperglycemia can lead to placental vascular resistance (34) and induce fetal hypoxia (35). Fetal hypoxia damages the hippocampus (36)—involved in behavioral and cognitive functions (35)—potentially causing developmental delay. Additionally, maternal chronic hyperglycemia may result in the development of microangiopathy or endothelial dysfunction (18). These symptoms lead a deficiency of nutrients and oxygen supply in a fetus, which leads to fetal growth restriction (37). It is associated with preterm birth and low birth weight (36), which are risk factors for ASD or ADHD (38). A study that examined the association between maternal type 1 DM and ASD in children also reported that approximately 20% of the total risk of ASD was mediated by preterm birth (39). Second, maternal hyperglycemia is associated with hyperglycemia in the fetus, and high glucose concentrations in utero can induce fetal hyperinsulinemia. Once delivered, a child cannot quickly reduce insulin secretion, which may cause fetal hypoglycemia (40). Fetal hypoglycemia may damage the brain and cause cognitive developmental delays (41). Moreover, hyperglycemia and hyperinsulinemia in a fetus lead to an increase of glucose uptake through glucose transporter 4, and it turns into fatty acid through increased expression of fatty acid synthase and acetyl coenzyme A carboxylase (42). This may lead to macrosomia (18), and high birth weight is associated with overweight (43), which may cause an increase of insulin resistance (43) and an increased risk of type 2 DM in the future (44). The previous study showed a volume of cerebral white matter was reduced in the whole brain and frontal lobe among obese adolescents with type 2 DM compared with those without type 2 DM (45). Thus, the functional vascular changes induced by type 2 DM may influence cognitive performance (45). Third, maternal hyperglycemia may increase reactive oxygen species production, inducing more oxidative stress in the cord blood, placental tissue (40), cerebral cortex, and hippocampus (46), which can affect neurodevelopment in the fetus (47). Regarding the timing of the exposure, the brain and central nervous system are established at 8 weeks of gestation (33), and the auditory network, which is a part of language skills, also starts developing from 8 weeks of gestation (48); therefore, the impact of hyperglycemia may occur during early pregnancy to 24 weeks of gestation.

In the present study, mFPG level ≤70 mg/dL was significantly associated with developmental delay across 5 domains in children. To the best of our knowledge, there is no study examining the association between maternal low blood glucose level and developmental delay in children. The possible reason for the significant association in the present study has been considered. It was possible for mothers who potentially had a high risk of DM or GDM to be included as a mFPG level 71 to 94 mg/dL, because mFPG level was only obtained at 1 point. Thus, it is possible to consider that a mFPG level ≤70 mg/dL included mothers with a genuine low risk of becoming DM or GDM. Therefore, a mFPG level ≤70 mg/dL significantly reduced the risk of developmental delay in children compared with a mFPG level 71 to 94 mg/dL. However, a previous study reported that a mFPG level ≤70 mg/dL during 24 to 28 weeks of gestation was associated with low birth weight in children (49). Low birth weight may associate with a risk of developmental delay (38); thus further study is needed to explore the influence on developmental delay in children.

The present study had some limitations. First, 6.5% of participants in the TMM BirThree Cohort Study were included in the present study. Variables obtained 2 years after delivery tended to have missing data, which results in a decrease in the follow-up rate, as reported in another cohort study in Japan (50). Few data on mFPG levels also influenced the number of participants included in the present study. Every hospital or clinic did not record the type of maternal blood glucose at the prenatal health check-ups. However, mFPG level before 24 weeks of gestation among participants who were excluded from the present study was not different compared with those who were included in the present study, as well as the prevalence of developmental delay (22). Second, the proportion of a mFPG ≤70 mg/dL was only 7.1% in the present study. A previous study reported that a mFPG level ≤75 mg/dL in the first trimester was 24.9% (51). In addition to few numbers of participants in the analysis, characteristics of people who participated to the cohort study may influence the proportion of a mFPG level ≤70 mg/dL. People who have high intentions to participate in the cohort study are likely to have healthier lifestyles, higher social status, and few histories of diseases (52). Thus, these characteristics may decrease risk factors of hypoglycemia such as malnutrition, alcohol addiction, or gastric resection. This may result in a decrease of the proportion of mFPG ≤70 mg/dL in the present study. The results may be underestimated in the present study. Third, the ASQ-3 is a nondiagnostic self-reported questionnaire answered by parents that may have led to misclassification; however, the original and Japanese versions of the ASQ-3 were validated (24, 25) and are commonly used in previous studies (12, 53, 54), and the present study could identify children who are at risk of having developmental disorders in the future. Finally, medication use was not considered in the present study. Mothers who were diagnosed with GDM after early gestation may have treatment. Lowered maternal blood glucose may decrease a risk of developmental delay in children, and the results might be underestimated.

Our findings found that mFPG level was not associated with an increased risk of developmental delay in children. Although a mFPG level ≤70 mg/dL was associated with developmental delay across 5 domains among children in the present study, further research is needed to examine the association between maternal hypoglycemia and developmental delay in children.

In conclusion, mFPG level in early gestation was not associated with an increased risk of developmental delay in children at the age of 2 years.

## Data Availability

The TMM BirThree Cohort Study data that support the findings of this study are not publicly available because they contain information that could compromise the participants’ consent. All inquiries regarding access to data should be sent to the TMM.
